# Nonradiative Deactivation of the Fluorescent Ag_16_-DNA and Ag_10_-DNA Emitters: The Role of Water

**DOI:** 10.1021/acs.jpclett.4c01959

**Published:** 2024-10-17

**Authors:** Ruslan R. Ramazanov, Rinat T. Nasibullin, Dage Sundholm, Theo Kurtén, Rashid R. Valiev

**Affiliations:** Department of Chemistry, University of Helsinki, P.O. Box 55 (A.I. Virtanens plats 1), University of Helsinki, FIN-00014, Finland

## Abstract

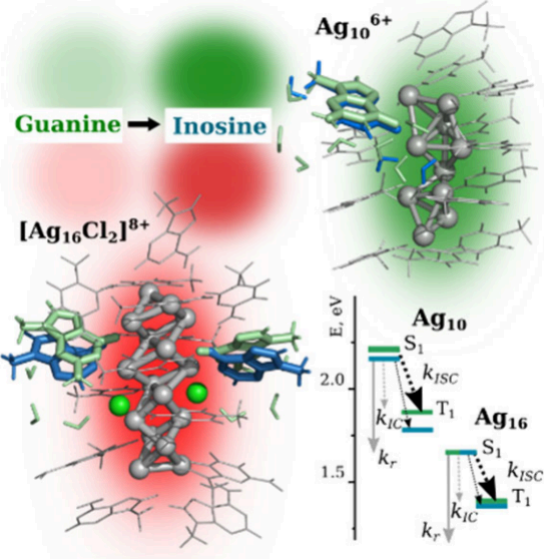

The luminescent quantum yield of silver-cluster emitters stabilized by short oligonucleotides (Ag_N_-DNA) may be efficiently tuned by replacing nucleobases in their stabilization DNA matrices with analogues. In the present study, we proposed a valuable and straightforward theoretical methodology for assessing the photophysical behaviors emerging in Ag_N_-DNA emitters after excitation. Using green Ag_10_-DNA and near-IR Ag_16_-DNA emitters we demonstrate how point guanine/inosine replacement could affect the photophysical rate constants of radiative/nonradiative processes. The main deactivation channel of the fluorescence of Ag_16_-DNA is intersystem crossing, which is in line with experimental data, whereas for Ag_10_-DNA the calculations overestimate the intersystem crossing rate possibly due to pure solvent contributions.

Silver clusters consisting of several tens of atoms and stabilized by short oligonucleotides (Ag_N_-DNA) are considered as promising quantum dots in biolabeling and biosensing, since their optical characteristics differ significantly from those of organic dyes.^1^ Besides greatly reduced bleaching and blinking as compared to existing organic dyes, they possess a significant quantum yield for forming long-lived dark states, which are highly demanded in optically activated delayed fluorescence microscopy.^2^ There is no clear understanding of the cluster formation processes yet. It is known that nucleotides form a stabilizing shell around groups of silver ions, which are then reduced by BH_4_ forming partially oxidized metal clusters.^3−6^ The size of the resulting clusters is determined by the number of available binding sites of the silver ions. Deprotonated nitrogen atoms of nucleotide heterocycles in the affinity series guanine(G) > cytosine(C) > adenine(A) ≫ thymine(T) are the most favorable binding sites.^7^ The shape of the clusters is defined by a cavity volume protected from external water solvent, formed by a flexible sugar–phosphate DNA backbone bonded with hydrophobic nitrogenous bases. A few heterogeneous Ag_N_-DNAs are formed in solution, which differ in charge, number of silver atoms and their ability to luminesce. Using HPLC-MS in combination with UV/vis and fluorescence spectroscopy, it is possible to separate bright luminescent structures of interest.^8^ The valence-band electronic structure of such separated optically active Ag_N_-DNA resembles that of the electronic structure of organic dyes, which have a highly intense long-wave absorption band corresponding to bright fluorescence in the visible and near-IR ranges.^9^

According to numerous steady-state and time-resolved spectroscopy data, fluorescence directly depends on the DNA sequence and the number of reduced metal atoms within the silver associates.^3,8,10−12^ It is possible to routinely vary the luminescent properties by selecting the required DNA matrix and the experimental conditions of the reduction of silver ions.^3,11^ An important feature has been revealed in mass spectrometry for many Ag_N_-DNA emitters - the number of pairs of confined 5s electrons of reduced silver atoms corresponds to a certain emission range in the fluorescence spectrum.^6^ For example, four confined electrons mainly correspond to the green emission range, and six confined electrons correspond to the red and near-IR range. This sorting of DNA matrices into color classes depending on the so-called “magic number” properties has made it possible to construct a supervised machine learning model that is able to determine how DNA sequence encodes Ag_N_-DNA color class.^13,14^ However, this approach is challenging to apply when trying to investigate the principles that determine the photophysical characteristics of Ag_N_-DNA emitters at the submolecular level.

The theory of the photophysical processes of the Ag_N_-DNA complexes is poorly developed since little is known about the atomic geometry of the clusters, which are stabilized by nucleotides. The most reasonable approach to delve into the nature of the photophysical features of luminescent emitters is a comparative consideration of the rates of radiative/nonradiative processes using time-resolved spectroscopy and methods of theoretical photophysics. In the last case, precise information is required about the atomic geometry and charge of the emitter under conditions in which the fluorescence is observed. Until recently, thinking about the shape and size of clusters was based on generalizing data from solving the inverse problem. A questionable identification of cluster structures is achieved when comparing the experimental luminescence excitation spectra with the excitation spectra of silver atomic geometries constructed by means of QM theory using indirect data on the number of atoms and the charge of clusters from mass spectrum.^15−17^

However, recently Vosch and Kondo^18^ succeeded in crystallizing luminescent Ag_N_-DNA in a X-ray study, which revealed the intricate structure of the near-IR emitter Ag_16_-DNA based on 16 silver atoms stabilized by two 5′-CACCTAGCGA-3′ sequences. The study has given a new impetus to theoretical studies. X-ray information about cluster geometry has boosted the interest of theorists in the possibility of understanding the details of the behavior of the excitation states of this emitter. Häkkinen et al.^19,20^ used real-space DFT calculations based on the projector-augmented wave method for optimizing the ground electronic state, the molecular structure as well as for calculating the absorption and CD spectra. The absorption spectrum was reproduced with high accuracy at the DFT level using the GLLB-SC functional and implicit solvent embedding.^19^ The later measured CD spectrum agrees well with the calculated one.^21^ Mikkelsen et al.^22^ studied the influence of the DNA environment on the optical properties of the Ag_16_ cluster using a time-dependent DFT framework combined with molecular mechanics via the polarizable embedding scheme. It has been shown that the main contributor to the observed emission is the silver cluster, while the DNA affects the optical response of the encapsulated silver cluster through the stabilization of an elongated geometry and Ag-DNA interaction. It should be noted that the geometry of the entire Ag_16_-DNA complex is practically not affected by crystallization since the experimental excitation and fluorescence spectra in the solution resemble those obtained in the crystalline phase.^18^ This makes it possible to consider the electronically excited structure of the Ag_16_-DNA within the framework of QM methods in isolated space without accounting for the restrictions imposed by the crystal cell.

Based on X-ray data for the near-IR-emitting Ag_16_-DNA complex, we computationally studied the (DNA)_2_[Ag_16_Cl_2_]^8+^ complex with 16 Ag atoms having a total charge of +10 bound to two Cl^–^ anions. We generalized the developed computational protocol to other clusters and carried out a comparative analysis of the structural features that determine the differences in the rates of radiative/nonradiative processes and luminescence quantum yields.

For comparison, we considered the luminescent Ag_N_-DNA complex, for which there is no resolved crystallographic structure. We chose the green Ag_10_-DNA emitter, which is well-studied by Petty et al.,^23^ containing a cluster of 10 silver atoms with a charge of +6, stabilized by a single oligomer 5′-C_4_AC_4_TC_3_GT_4_-3′. We reconstructed the atomistic structure of Ag_10_-DNA based on the assumption that each silver atom of the cluster must be associated with at least one nitrogenous base, primarily cytosine or guanine. We bent the 5′-C_4_AC_4_TC_3_GT_4_-3′ sequence in this way so that the condition for the silver atoms binding by cytosines and guanines was satisfied while the accessibility of the solvent into the cavity was minimized. Based on Ag_16_-DNA crystallography data, we assumed that adenines and thymines can act as flexible elements and promote chain bending, while cytosines and guanines are predominantly associated with silver and are stabilized by stacking interactions with each other.

In addition, we assessed changes in the fluorescence quantum yields of the selected Ag_16_-DNA and Ag_10_-DNA emitters, which emerge because of point guanine/inosine mutations. In recent experimental studies, the substitution of guanines for inosines in DNA structures resulted in an increase of fluorescence quantum yield from 25% to 35% for Ag_16_-DNA^24^ and from 25% to 63% for Ag_10_-DNA.^25^ Since inosine is more hydrophobic than guanine due to the absence of an amino group at the C_2_ position, one could expect the different effects of explicitly specified water molecules on calculated photophysical properties. It should also be noted that G_9_ and I_9_ in the Ag_16_-DNA structure are bonded to the silver cluster via a deprotonated N_1_ atom. Deprotonation of guanines upon binding to silver ions or clusters has been observed previously.^26^ Silver has been shown to reduce the p*K*_a_ value of guanine at the N_1_ position, as well as the p*K*_a_ of inosine, which can lead to deprotonation and formation of a N_1_–Ag bond.^27^ Zhang’s et al.^28^ recent time-resolved study showed that in the Ag_10_-DNA structure, guanine and inosine are in the protonated state, and binding with silver can occur only at the N_7_ and O_6_ atoms. We used this assumption when reconstructing our Ag_10_-DNA model. When guanine G_9_ is replaced in the structure of Ag_16_(G) with the sequence 5′-CACCTAGCGA-3′ by inosine, the Ag_16_(I) structure with the sequence 5′-CACCTAGCIA-3′ is obtained. When guanine G_14_ is replaced in the Ag_10_(G) structure with the sequence 5′-C_4_AC_4_TC_3_GT_4_-3′ by inosine, the Ag_10_(I) structure with the sequence 5′-C_4_AC_4_TC_3_IT_4_-3′ is obtained. All studied complexes are illustrated in Figure 1.

**Figure 1:**
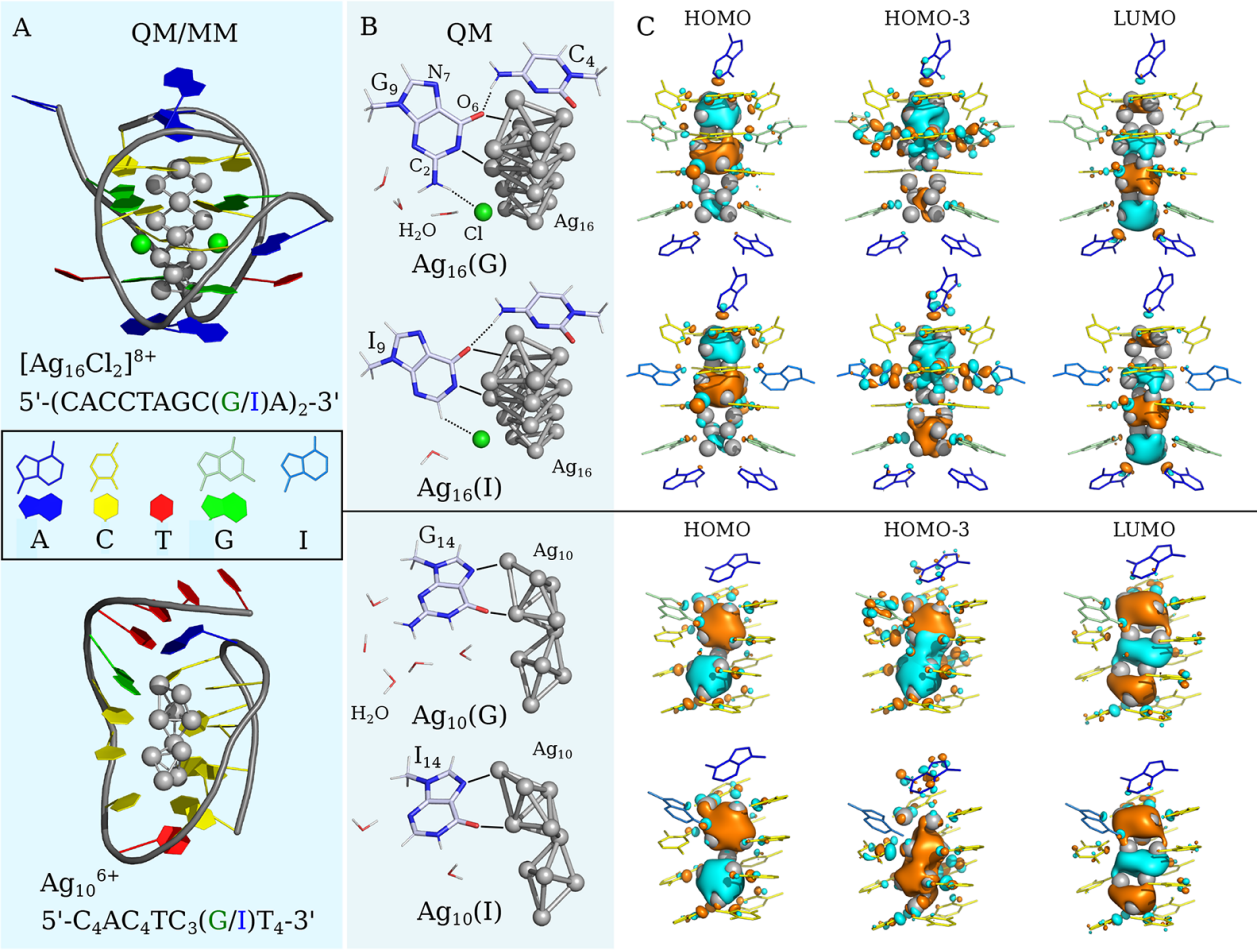
Illustration of (A) the QM/MM optimized *S*_0_ molecular structure of the full-length DNA matrices, including a sugar–phosphate backbone and nucleotides bound to a silver cluster and (B) the QM optimized *S*_1_ molecular structure of a truncated system consisting of a silver cluster, nucleotides with attached methyl groups instead of ribose residues and explicit water molecules near the guanines/inosines and (C) the corresponding HOMO, HOMO–3, and LUMO orbitals participating in the *S*_1_ → *S*_0_ transition with a contour threshold value of 0.02 au.

By summarizing computational modeling steps based on the modeling of the X-ray structure of Ag_16_-DNA, a protocol for calculating the equilibrium geometries of the *S*_0_ ground state and the *S*_1_ excited state of Ag_N_-DNA complexes can be formulated. In doing so for Ag_16_-DNA with the guanine/inosine replacement, we used the starting geometry from the crystallographic data reported by Cerretani et al. (PDB ID: 6JR4).^18^ Qualitative validation of the calculated equilibrium geometries was carried out by comparing the absorption and fluorescence spectra calculated using the optimized geometries and those obtained in experiments. The vibrational spectra calculated for the *S*_0_ and *S*_1_ optimized geometries of the DNA-cluster complex did not contain any imaginary frequencies indicating that local minima on the potential energy surface have been reached in the structure optimizations. Details of the QM and QM/MM calculations of the Ag_N_-DNA geometries are described in the Supporting Information (SI).

To estimate the quantum yield of fluorescence (QYF), fast algorithms for calculating the rate constants of radiative/nonradiative processes were used.^29−31^ The correct theoretical assessment of the competition between different photophysical processes is a challenging task that is associated with the correctness of the adopted structural and photophysical models. In earlier studies of silver clusters stabilized by DNA, one concluded that nonradiative processes mainly occur through internal conversion to the ground state or to a long-lived dark state. Several alternative ways of exciton relaxation were shown in a study by Patel et al.^32^ who performed transient absorption (TA) measurements of silver clusters stabilized by single stranded DNA (ssDNA) on different time scales of up to tens of microseconds. They found that the exciton rapidly relaxes via a recombination process to the ground state suggesting that internal conversion is the main quenching channel of the fluorescence. Another long-lived dark state with a lifetime of ∼10 μs and a quantum yield of formation of about 1% was observed. Zhang et al.^25^ showed that the quantum yield of the formation of the dark state of the Ag_10_^6+^ cluster stabilized by 5′-C_4_AC_4_TC_3_GT_4_-3′, which is called Ag_10_(G) in the present study, is less than 4%, while in the TA experiments a significant repopulation of the ground state was observed suggesting that internal conversion is the main channel quenching the fluorescence. Chen et al.^33^ did not observe any ground-state recovery on the fluorescence lifetime scale in the TA experiment on the Ag_16_^10+^ cluster stabilized by 5′-CACCTAGCGA-3′, which is called Ag_16_(G) in the present study, suggesting that the relaxation mainly occurs via a long-lived dark state rather than directly to the ground state via internal conversion. Thus, the nonradiative exciton relaxation may indeed have different dominant channels for different DNA stabilized fluorescent clusters.

Despite some TA experiments that have convincingly demonstrated the formation of dark states competing with the fluorescence, the nature of these dark states has been discussed. Copp et al.^34^ showed that the Lippert-Mataga model is not applicable to Ag_N_-DNA systems when considering the solvatochromism arising in ethanol–water and methanol–water mixtures. They stated that the solvatochromic behavior of Ag_N_-DNA provides no evidence for the formation of charge transfer states after excitation, as was suggested before.^32^ The idea that the dark state could be a charge-transfer state was not supported. The conformation of the secondary structure of the DNA changes when adding alcohol, which is the well-known conformational B-A transition.^35^ The observed change in the solvatochromic shift is not due to solvent sensitivity of the charge-transfer state but is probably caused by the influence of the water–alcohol mixture on the packing of the single-stranded DNA sequence, which affects the geometry of the silver cluster or their excited state properties. By using D_2_O instead of H_2_O as solvent, Cerretani et al.^36^ showed in a study on the Ag_16_^10+^ cluster stabilized by 5′-CACCTAGCGA-3′ that the formation of a μs-lived state is enhanced, which leads to a lengthening of the ⟨*τ*_*μs*_⟩ time as a triplet state is formed via intersystem crossing. Rück et al.^37^ also reported an almost 2-fold extension of the lifetime of the μs-lived luminescent state of DNA_2_[Ag_18_]^12+^ when using D_2_O rather than H_2_O as solvent. The triplet state is most likely the dark state.

In our photophysical model, we consider the triplet state as the dark state, which depends mostly on the inner electronic properties of the cluster surrounded by the DNA matrix. We suggest that the main processes that compete with the fluorescence are nonradiative internal conversion (*S*_1_ → *S*_0_) and intersystem crossing (*S*_1_ → *T*_1_). Calculations of the internal conversion (IC) rate constants (*k*_*IC*_) of the *S*_1_ → *S*_0_ transition processes, intersystem crossing (ISC) rate constants (*k*_*ISC*_) of the *S*_1_ → *T*_1_ transition processes, as well as the radiative rate constants (*k*_*r*_) of the *S*_1_ → *S*_0_ transition processes were carried out using the optimized molecular structure of the *S*_1_ state of the Ag_N_-DNA complexes by performing QM calculations of spin–orbit coupling matrix elements (SOCME), nonadiabatic coupling matrix elements (NACME), excitation energies of the singlet *S*_1_ state and the lower-lying triplet states. Details of the theory for calculating photophysical rate constants are described in the SI.

A general protocol for studying the photophysical parameters of Ag_N_-DNA complexes consists of several stages (illustrated in Figure S1):

(a) A full-length DNA matrix, including a sugar–phosphate backbone and nucleotides, in combination with a silver cluster, is placed in an aqueous cell, in which QM/MM optimization using adapted plane-wave type DFT^16,38,39^ of the QM part of the ground state geometry is performed.
(b) A truncated system including the silver cluster, nucleotides with methyl groups instead of ribose residues, and explicitly defined water molecules near the guanines/inosines of interest are placed in a continuum aqueous environment. Sequential QM optimization at the DFT level using Gaussian type basis functions is carried out for the geometry of the ground state and for the *S*_1_ excited state, with the constraint that the carbon atoms of the methyl groups are fixed. Fixing the carbon atoms of the methyl groups simulates the low mobility of the sugar–phosphate backbone during the relaxation of the molecular structure of the *S*_1_ state.
(c) For the optimized molecular structure of the ground state, the absorption spectra are calculated at the time-dependent DFT level. The computational methods are described in detail in the SI. For the optimized molecular structure of the *S*_1_ state, we calculate the excitation energies and oscillator strength of the lowest singlet state yielding the fluorescence spectra, the excitation energies of the lowest triplet excited states, as well as SOCME and NACME.

After a preliminary QM/MM optimization of the silver cluster and the full-length DNA matrices, including the sugar–phosphate backbone and nucleotides, we studied truncated complexes consisting of the silver cluster and nucleotides with attached methyl groups instead of ribose residues. The position of the carbon atoms of the methyl groups were fixed to simulate the low mobility of the sugar–phosphate backbone. Since negatively charged DNA phosphates repel each other in water solution regulating the DNA folding,^40,41^ removing the sugar–phosphate backbone and freezing the corresponding carbon atoms of the methyl groups leads to a very small relaxation of the backbone when optimizing the molecular structure of the ground and the first excited singlet (*S*_1_) states employing an implicit solvent continuum model. Single-point energy calculations and the geometry optimization of the excited states were performed on silver cluster surrounded by the nitrogenous bases. It has previously been shown that implicit solvent models based on the Onsager reaction field may not accurately describe the solvatochromism of the Ag_N_-DNA emission observed in water–alcohol mixed solvents.^34^ Here, we investigated how well our model can qualitatively describe the photophysical properties of silver clusters stabilized by short DNA strands.

The molecular structure of the ground state of truncated complexes was calculated at the PBE0/def2-TZVP level using the PCM solvation model. The obtained structure of Ag_16_(G) resembles the X-ray structure with an RMSD value of 5.78 Å when considering all atoms and an RMSD value of 3.78 Å when considering only the 16 Ag atoms and the two Cl atoms (Table S1). The superimposed X-ray structure on the optimized ground state structure of Ag_16_(G) is shown in Figure S2 B. Despite the molecular structures slightly differ, the calculated excitation spectrum is in good agreement with the experimental spectrum measured in solution. The largest differences between calculated and experimental structures of the *S*_0_ state are about 0.4 Å for the Ag–Cl bonds and about 0.2 Å for the Ag–Ag bonds. The Ag–Cl and Ag–Ag bond lengths are reported in Table S2.

The optimized molecular structure of the *S*_1_ state of Ag_16_(G) differs only slightly from the ground state geometry. The superimposed structures are shown in Figure S2 C. The RMSD value is 0.16 Å for the 16 Ag atoms and two Cl atoms. The two structures differ significantly with an RMSD value of 7.78 Å when considering all atoms (Table S1) showing that the main relaxation occurs at the nucleotides. The *S*_1_ geometry of Ag_10_(G) differs only slightly from the molecular structure of the ground state. The superimposed structures are shown in Figure S3 A. The RMSD value is 4.41 Å when all atoms are considered and 0.08 Å when only the 10 Ag atoms are included (Table S3). The guanine/inosine substitution of Ag_16_-DNA leads to only a small change in the *S*_0_ and *S*_1_geometries (Figure S2, D), whereas inosine is shifted relative to guanine as also observed in the X-ray structures.^24^

For the optimized molecular structures of the *S*_0_and *S*_1_states of the studied complexes, the absorption and fluorescence spectra were calculated at the TD-DFT level. The calculated spectra of Ag_10_(G) and Ag_16_(G) are compared to the experimental ones in Figure 2.

**Figure 2:**
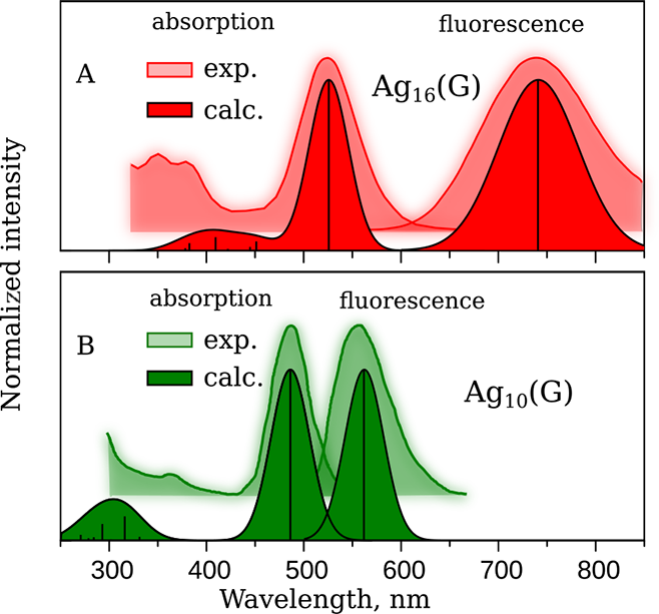
Comparison of the experimental absorption and fluorescence spectra of Ag_10_(G)^42^ and Ag_16_(G)^33^ with the ones calculated at the TDDFT level.

The excitation energies of the triplet states for the ground state geometries are very little affected by the substitution of guanine with inosine. For the ground state (*S*_0_) geometries of Ag_16_-DNA complexes, we obtained two triplet states below the *S*_1_ state (Table 1). For the *S*_0_ geometries of Ag_10_-DNA complexes we obtained only one triplet state below the *S*_1_ state. The first *T*_1_ state of the Ag_16_-DNA complex is dominated by the HOMO → LUMO transition (Table S4). The *T*_2_ state, which is nearly degenerate with the *S*_1_ state, is dominated by the HOMO → (LUMO+1) transition, where the LUMO+1 orbital is mainly on the silver atoms close to the pair of A_6_ adenines and the two chlorines (Figure S4). The *T*_1_ state of the Ag_10_-DNA complex is also dominated by the HOMO → LUMO transition, which is mainly localized on the cluster (Figure S5).

**Table 1:** Comparison of the Experimental and Calculated Excitation Energies (in eV) of the Lowest Singlet and Triplet Excited States, the Spin–Orbit Coupling Matrix Elements (⟨*S*_1_ | *H*_*SO*_ | *T*_1_⟩ in cm^–1^), the Radiative Rate Constants*k*_*r*_ (s^–1^), the Intersystem Crossing Rate Constants*k*_*ISC*_ (s^–1^), the Internal Conversion Rate Constants*k*_*IC*_ (s^–1^), and the Quantum Yield of Fluorescence QYF (%)^a^

Optimized molecular structure	Electronic state/property		Ag_16_(G)	Ag_16_(I)	Ag_10_(G)	Ag_10_(I)
*S*_0_	*T*_1_		1.98	1.97	1.95	1.95
	*T*_2_		2.31	2.30	–	–
	*S*_1_	calc.	2.36 (0.87)	2.37 (0.91)	2.55 (0.79)	2.57 (0.79)
		exp.	2.36^b^	2.36^b^	2.53^c^	2.53^c^
*S*_1_	*T*_1_		1.44	1.43	1.84	1.78
	*S*_1_	calc.	1.67 (1.33)	1.66 (1.27)	2.2 (1.19)	2.17 (1.21)
		exp.	1.68^b^	1.70^b^	2.18^c^	2.17^c^
	⟨*S*_1_ | *H*_*SO*_ | *T*_1_⟩		3.01	2.05	4.09	3.68
	*k*_*r*_(*S*_1_ → *S*_0_)		1.61 × 10^8^	1.54 × 10^8^	2.49 × 10^8^	2.47 × 10^8^
	*k*_*ISC*_(*S*_1_ → *T*_1_)		1.96 × 10^9^	8.25 × 10^8^	8.79 × 10^8^	4.85 × 10^8^
	*k*_*IC*_(*S*_1_ → *S*_0_)		4.96 × 10^6^	4.96 × 10^6^	1 × 10^6^	1 × 10^6^
	QYF	calc.	8	16	22	34
		exp.	26^b^	36^b^	25^c^	63^c^

a Oscillator strengths are given in parentheses.

b Ref (24).

c Ref (25).

In the optimized molecular structure of the *S*_1_ state of Ag_10_-DNA and Ag_16_-DNA, only *T*_1_ lies below the *S*_1_ state (Table 1) implying that the rate constant of the ISC process from the *S*_1_ state is determined by the probability of the transition to *T*_1_ and depends on the size of the *S*_1_ → *T*_1_ energy difference and the strength of the spin–orbit coupling between *S*_1_ and *T*_1_. The calculated energies of the *S*_1_ states in the relaxed *S*_1_ geometries correspond well to the experimental fluorescence wavelengths with a deviation of less than 0.04 eV (Table 1).

For estimating the QYF, it was necessary to assess to what extent the radiative process competes with nonradiative *S*_1_ relaxation processes, such as the IC (*S*_1_ → *S*_0_) and ISC (*S*_1_ → *T*_1_). The radiative rate constants (*k*_*r*_) were calculated using the Strickler–Berg formula.^43^ Due to the significant intensity of the low-lying transitions and the relatively small energies of the *S*_1_ states of the studied complexes, we obtained large *k*_*r*_ values on the order of ∼10^8^ s^–1^. The calculated radiative rate constants correspond to the experimentally observed fluorescence lifetimes on the nanosecond time scale.^44,45^ The calculated *k*_*r*_ of 1.61 × 10^8^ s^–1^ for Ag_16_(G) agrees well with the experimental one of 7.9 × 10^7^ s^–1^.^33^ For Ag_10_(G), the calculated *k*_*r*_ of 2.49 × 10^8^ s^–1^ also agree well with the experimental one of 8 × 10^8^ s^–1^.^46^ Despite the large size of the Ag_N_-DNA complexes, the HOMO and LUMO orbitals corresponding to the *S*_1_ → *S*_0_ transition are almost completely localized on the silver cluster (Figure 1 C). The HOMO and LUMO orbitals for Ag_16_-DNA complexes indicate that the two chlorines participate in the collective excitation of silver atoms with their n-orbitals. The *S*_1_ → *S*_0_ transition has a large oscillator strength because of the local character of the transition.

It should be noted that in the *S*_1_ state, in addition to the HOMO–LUMO transition with a configuration interaction (CI) coefficient of 0.7, there are also transitions from HOMO–3 to LUMO and to higher-lying LUMO orbitals with a total contribution of less than 0.03 (Table S5). Each of the rest transitions forming the *S*_1_ state was omitted from consideration since they made negligible contributions, less than 0.02. In the case of Ag_16_(G) and Ag_16_(I), the HOMO–3 orbitals mix the π-orbitals of G_9_ or I_9_ with the orbitals of the confined 5s electrons of the silver cluster (Figure 1 C). In the case of Ag_10_-DNA, only the HOMO–3 orbitals of Ag_10_(G) mix the π-orbitals of G_14_ with the orbitals of the confined 5s electrons of the cluster. However, due to the small contribution from these orbitals to the *S*_1_ state, they practically do not affect the local character of the *S*_1_ → *S*_0_ transition and have no effect on the high probability of the transition.

To estimate the ISC rate constants *k*_*ISC*_, the energies of triplet states below the *S*_1_ state, as well as the SOCME values for the corresponding *S*_1_ and *T*_1_ states in the relaxed *S*_1_ geometries were calculated. In the case of replacing guanine with inosine in the Ag_16_-DNA complex, we did not observe any significant differences in the energies of the *S*_1_ and *T*_1_ states (Table 1). There are three water molecules near guanine, and one water molecule near inosine. In the *T*_1_ state, just like in *S*_1_, in addition to the HOMO–LUMO transition with a CI coefficient of 0.7, there are transitions from HOMO–3 to LUMO and higher-lying LUMO orbitals, but with a total contribution of 0.1, which is greater than 0.03 for *S*_1_ (Table S5). Each of the rest transitions forming the *S*_1_ state was omitted from consideration since they made negligible contributions, less than 0.02. It could be expected that due to the greater contribution from the HOMO–3 orbital to the *T*_1_ state, under conditions with different numbers of water molecules around guanine and inosine, they would have a different effect on the energy of the *T*_1_ state than on the energy of the *S*_1_ state. However, the decrease in *S*_1_ and *T*_1_ energies by 0.01 eV in Ag_16_(I) relative to Ag_16_(G) are much smaller than uncertainties in the DFT energies. The calculated SOC values for Ag_16_(G) of 3.01 cm^–1^ are greater than the SOC for Ag_16_(I) of 2.05 cm^–1^, and the *k*_*ISC*_ = 1.96 × 10^9^ s^–1^ for Ag_16_(G) is twice greater than *k*_*ISC*_ = 8.25 × 10^8^ s^–1^ for Ag_16_(I). Figure 1 B shows that in the optimized *S*_1_ geometry, the guanine forms a stabilizing hydrogen bond C=O···NH_2_ with a length of 2.72 Å with the nearby cytosine C_4_ and an electrostatic NH_2_–Cl bond with a length of 3.41 Å, while a C=O···Ag bond with a length of 2.34 Å is formed. After geometry relaxation of the inosine complex, the C=O···NH_2_ hydrogen bond becomes 3.2 Å longer and an electrostatic CH-Cl bond is formed with a length of 3.61 Å, while the C=O···Ag distance increases to 2.92 Å. It is known that carbonyl groups can influence the SOC value regulating the ISC process.^47^ The difference in the *S*_1_ geometry relaxation of guanine and inosine complexes with silver cluster resulted in alternating size of the *k*_*ISC*_ values. Chen et al.^33^ showed that the relaxation of the excited *S*_1_ state of Ag_16_(G) must mainly occur via ISC (*S*_1_ → *T*_1_) rather than directly to the ground state via IC (*S*_1_ → *S*_0_). The present calculations using the optimized molecular structure of the *S*_1_ state of Ag_16_(G) and Ag_16_(I) are consistent with these data, since *k*_*ISC*_(Ag_16_-DNA) > *k*_*R*_(Ag_16_-DNA) implying that ISC quenches the fluorescence.

In the case of replacing guanine with inosine in the Ag_10_-DNA complex, we observe a decrease in the energy of the *S*_1_ state by 0.03 eV, and the energy of *T*_1_ decreases by 0.06 eV. There are five water molecules near guanine, and two water molecules near inosine. Careful examination of the location of guanine and inosine relative to the silver cluster did not reveal any significant differences. Like for Ag_16_-DNA, we observed for the *T*_1_ state of the Ag_10_-DNA structures that in addition to the HOMO–LUMO transition, the contribution of the transition from HOMO–3 orbital to LUMO and higher-lying LUMO orbitals with a net CI coefficient of 0.1 is greater than 0.03 for the *S*_1_ state (Table S5). However, analysis of the HOMO–3 orbital revealed that for Ag_10_(I), the π-orbital of inosine does not mix with the cluster orbitals, as occurs for guanine in Ag_10_(G). Apparently, under conditions of mixing of the guanine orbitals with cluster orbitals in HOMO–3, explicitly considered water molecules interacting in the *S*_1_ state with the amino group of guanine destabilizes the triplet state and shifts its energy to the blue region. Under conditions when inosine orbitals do not mix with the cluster orbitals in HOMO–3 water generally leads to stabilization of the triplet state and shifts its energy to the red region. The water influence also alters the SOC values. The calculated SOC value for Ag_10_(G) of 4.09 cm^–1^ is greater than the SOC value for Ag_16_(I) of 3.68 cm^–1^, and the *k*_*ISC*_ = 8.79 × 10^8^ s^–1^ for Ag_10_(G) is almost twice greater than *k*_*ISC*_ = 4.85 × 10^8^ s^–1^ for Ag_10_(I). The ISC rate constants of the Ag_10_-DNA complexes are overestimated by about one order of magnitude, since for Ag_10_(G) the experimentally estimated *k*_*ISC*_ is 5 × 10^7^ s^–1^.^46^

To assess the efficiency of the IC (*S*_1_ → *S*_0_) process, we used the approximation of promoting X-H vibrational modes using the approach developed by Valiev et al.,^29,48^ in which the electronic excitation energy is transformed into X-H (with X = C, O, N or another atom) vibrations since these bonds have large anharmonicity. This method can be applied to rather large molecules, even though it is computationally expensive. The method has been verified and applied to various types of molecules and molecular complexes, including organometallic compounds, for which IC and energy transfer were calculated.^31^

Dissipation of the excitation energy to heat through fast anharmonic vibrations of the X-H bonds of the DNA fragments is efficient when the molecular orbitals involved in the *S*_1_ → *S*_0_ transition are located on atoms involved in these vibrational modes. Since the orbitals of the *S*_1_ → *S*_0_ transition are almost completely localized on the silver cluster with small contribution from the orbitals at the X-H bonds of the nucleotides (guanine/inosine), the *k*_*IC*_ calculated in the X-H bond approximation is ∼10^6^ s^–1^, which is several orders of magnitude smaller than *k*_*r*_ (Table 1). While our photophysical model considers mainly the intrinsic vibrational relaxation channels of the Ag-DNA complex, the previously proposed dynamic quenching mechanism involving the amino group of guanine seems to be less important.^24^ According to our calculations, the IC process cannot compete with the radiative transition, which agrees with the experimental data for Ag_16_-DNA, but to some extent contradicts the experimental data for Ag_10_-DNA. On the other hand, the *k*_*ISC*_ rate constants are in general greater than *k*_*r*_. The calculations suggest that the ISC process competes with the fluorescence transition, which are thus the main relaxation channels of the fluorescent *S*_1_ state of Ag_16_-DNA and Ag_10_-DNA.

The calculated ratios of the QYF of the guanine/inosine substituted Ag_16_-DNA complexes agree qualitatively with the experimental ones (Table 1). The underestimated QYF may be due to a systematic overestimation of *k*_*ISC*_. Considering solvent effects from explicit water molecules is expected to increase the energy gap between the *S*_1_ and *T*_1_ states, which decreases *k*_*ISC*_ and leads to a better agreement between calculated and measured QYF.

Similar calculations overestimate *k*_*ISC*_ and underestimate *k*_*IC*_ for Ag_10_-DNA leading to a QYF that does not agree well with experimental data. Despite the excellent agreement between calculated and measured steady-state spectroscopy data, omitting the influence of the water molecules leads to inaccurate values for rate constants of the nonradiative transitions.

The relaxation of the molecular structure of the *S*_1_ state of the silver clusters embedded in the DNA matrix may significantly depend on explicit water molecules, which stabilize the secondary structure of oligonucleotides. Penetration of water molecules into the DNA moiety also results in a smaller density of the DNA packing around the cluster. It can be assumed that in the more compact Ag_16_-DNA structure, water access to the cluster is more limited than in the flexible and less compact Ag_10_-DNA structure, which may be the main reason for the differences in the *k*_*ISC*_ and *k*_*IC*_ values of the studied Ag_N_-DNA complexes. Thus, the use of the more extended explicit water model seems to be essential.

A recent X-ray study of Ag_11_-DNA by Rück et al.^49^ showed that it has a similar structure as the optimized one for Ag_10_-DNA. They have also very similar absorption and luminescence spectra in solution. The Ag_11_-DNA study shows that the molecular structure is very soft because crystallization significantly changes it. To elucidate photophysical properties of DNA-stabilized silver clusters based on crystallographic data, it is necessary to use modeling tools that correctly describe the effect of the water molecules on the folding of DNA-silver complex and the excitation energies. Possible nonradiative relaxation pathways caused by interactions between water molecules and silver atoms must be considered.

We have developed a computational approach and showed its applicability for modeling photophysical properties of Ag_N_-DNA fluorophores consisting of more than one hundred atoms. Obviously, further consideration of the details of the DNA packing in explicit solvent is required. Applying the methods to DNA-stabilized silver clusters, with or without using crystallographic data, will significantly advance our understanding of the nature of photophysical phenomena of this class of emitters.

## Supporting Information Available

Additional information regarding the applied photophysical methods and supplementary figures and tables (PDF)
