# Supplementary material for: Nonradiative Deactivation of the Fluorescent Ag16-DNA and Ag10-DNA Emitters: The Role of Water
Source: J Phys Chem Lett. 2024 Oct 17;15(42):10710–7. doi: 10.1021/acs.jpclett.4c01959 (PMC11514010; doi:10.1021/acs.jpclett.4c01959)
Supplement: Supplementary file 1 — jz4c01959_si_001.pdf [file jz4c01959_si_001.pdf]

Supporting Information

Nonradiative deactivation of the fluorescent Ag_{16}-DNA and Ag_{10}-DNA emitters: the role of water

Ruslan R. Ramazanov, Rinat T. Nasibullin, Dage Sundholm, Teo Kurtén and Rashid R. Valiev**

Department of Chemistry, University of Helsinki, P.O. Box 55 (A.I. Virtanens plats 1), University of Helsinki, FIN-00014, Finland.

Corresponding Authors

*R.R. Ramazanov: ramazarura@gmail.com, R.R. Valiev: valievrashid@gmail.com

Computational protocol

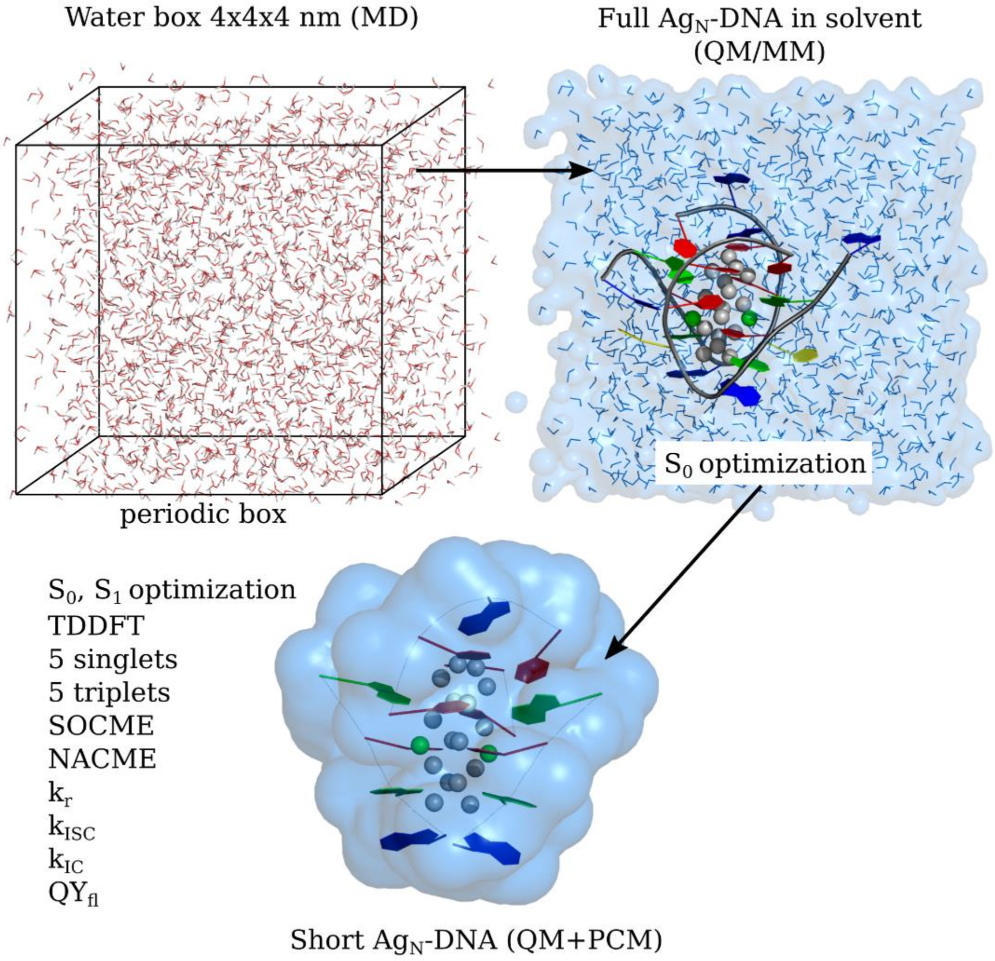

Figure S1. Illustration of all stages of the computational scheme.

- *Preparation of Ag_N-DNA in an aqueous environment*

We removed the Ag_{16} cluster surrounded by two 5′-CACCTAGCGA-3′ DNA strands from the crystal cell (PDB ID: 6JR4)[1] and placed it in a 4x4x4 nm aqueous cubic box. A water box containing 2165 water molecules was pre-equilibrated in a ten ns molecular dynamics simulation at 300 K in the NPT ensemble using the TIP3P water model under periodic boundary conditions using Gromacs2021.[2] The overlapping water molecules were removed when placing Ag_{16}(G) in the water cell.

- *Full-size system optimization at the QM/MM level*

Using QM/MM level in the CP2K program[3] the geometry of the $Ag_{16}(G)$ complex in an aqueous solution was optimized. In the QM/MM optimization of the ground state geometry, we used an additive Hamiltonian[4] including QM treatment of Ag-DNA complex and an MM description of the surrounding water molecules. The non-covalent interactions between the QM and MM parts were represented by electrostatic interaction using the Gaussian expansion of the electrostatic potential method (GEEP) employing the electrostatic coupling procedure.[4] The QM part used the DFT level in combination with the Gaussian and plane-wave (GPW) scheme[5] for representing the electron density, triple-zeta valence plus polarization (TZVP) basis sets of the MOLOPT[6,7] type to describe the valence electrons and norm-conserving Goedecker-Teter-Hutter (GTH)[8,9] pseudopotentials to approximate the core electrons, the PBE0[10] hybrid functional with Grimme's D3[11] dispersion correction to account for van der Waals interactions in QM part. To achieve acceptable performance when calculating with the hybrid DFT functional, we used an auxiliary density matrix method (ADMM)[12] and the corresponding triple-zeta auxiliary basis set. To map the DFT Gaussians onto the grids under the GPW representation we used a planewave cutoff of 700 Ry.

- *Optimization and excited state energy calculations at the QM level of the S_0 and S_1 states of a truncated molecular system*

After reaching the minimum gradient norm of 5×10^{-4} in the QM/MM optimization, the cluster structure surrounded by nitrogenous bases was cut out, and the ribose residues were replaced by methyl groups. The carbon atoms of the methyl groups were fixed in the final optimization of the molecular structure of the ground (S_0) and S_1 states that was carried out using the Gaussian16 program[13], the PBE0[10] functional, the PCM[14] continuum solvent model for water, the def2-SVP basis set with an effective pseudopotential considering the 28 core electrons of the silver atoms and the 6-31G* split-valence basis set[15] with polarizing functions on the organic atoms. In the geometry optimization, the water molecules taken from the optimized QM/MM structures were placed near guanines and inosines. We calculated the electronic excited states using the TDDFT approach and the PBE0 functional. The five lowest excited singlet and triplet states were calculated using the PCM continuum solvent model for water, the def2-TZVP[16] basis

set for the silver atoms and the 6-31+G* split-valence basis set with diffuse and polarizing functions on the organic atoms.

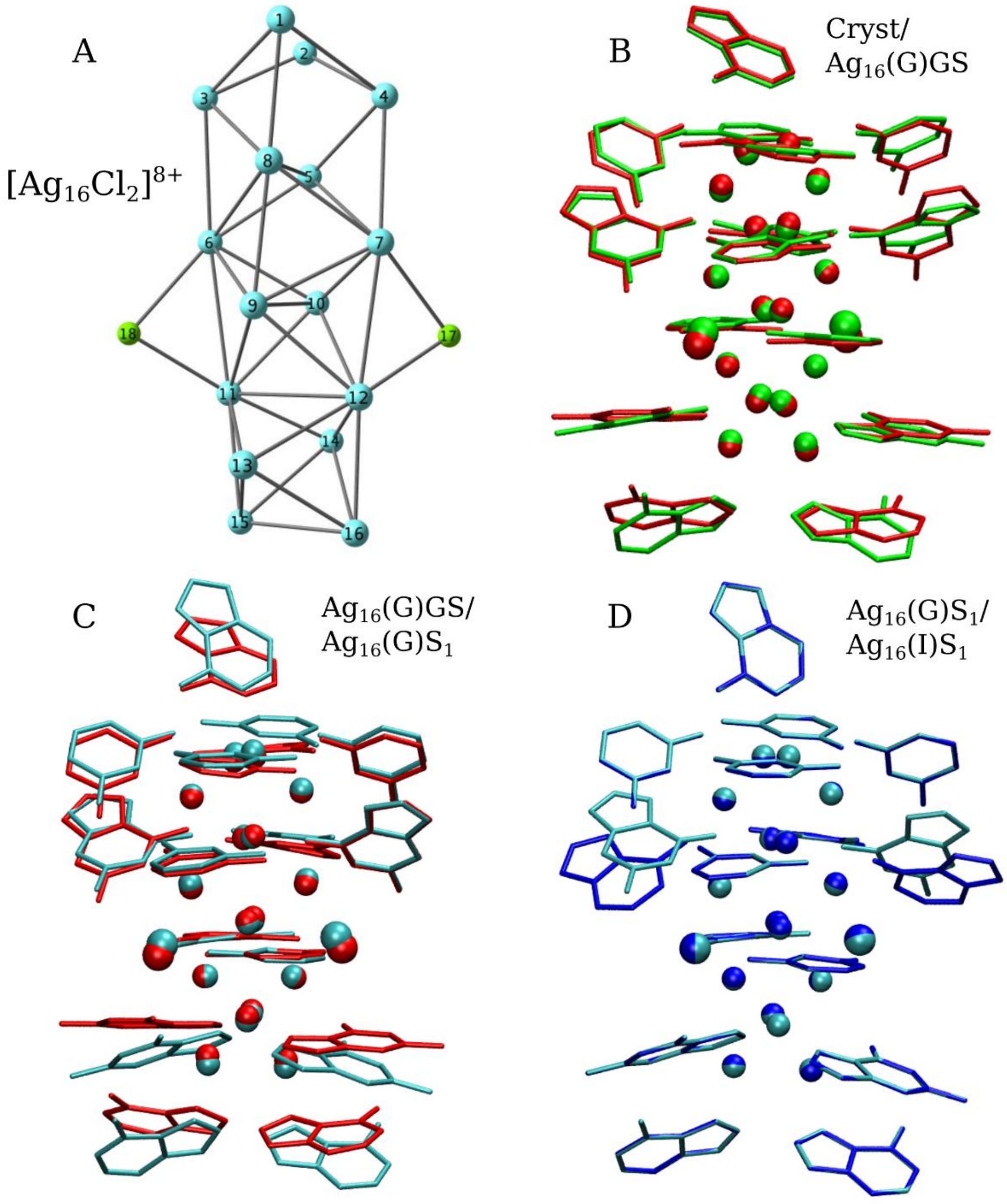

Figure S2. A) Illustration of the $[Ag_{16}Cl_2]^{8+}$ structure with numbering of all the atoms. B) Superimposed structures of the X-ray structure (green) with the molecular structure of C) the ground state (GS) of Ag_{16}(G) (red), the S_1 state of Ag_{16}(G) (cyan) and D) the S_1 state of Ag_{16}(G) (cyan) and the S_1 state of Ag_{16}(I) (blue).

Table S1. RMSD values of the superimposed X-ray, GS and S_1 geometries of Ag_{16}(G).

	Ag_{16} X-ray /Ag_{16}(G) S_0		Ag_{16}(G) S_0 / Ag_{16}(G) S_1	
	Ag_NCl_2	Full	Ag_NCl_2	Full
RMSD, Å	3.78	5.78	0.16	7.78

Table S2. Comparison of the Ag-Cl and Ag-Ag bonds of the X-ray and QM-optimized ground state geometries of the Ag_{16}(G). Table shows the bond lengths that not exceeded 3.5 Å. The numbers of atoms illustrated in the figure S2 A.

Å	N_1	N_2	Ag_{16} X-ray	Ag_{16}(G) S_0		N_1	N_2	Ag_{16} X-ray	Ag_{16}(G) S_0
Ag-Cl	12	17	2.56	2.55	Ag-Ag	14	16	2.79	2.87
Ag-Cl	11	18	2.60	2.55	Ag-Ag	11	15	2.80	2.99
Ag-Cl	7	17	2.71	3.15	Ag-Ag	13	15	2.80	2.84
Ag-Cl	6	18	2.75	3.21	Ag-Ag	1	2	2.83	2.84
Ag-Cl	9	18	3.01	3.36	Ag-Ag	9	12	2.83	2.99
Ag-Cl	10	17	3.07	3.41	Ag-Ag	9	11	2.84	2.95
Ag-Ag	7	10	2.69	2.76	Ag-Ag	2	5	2.85	2.85
Ag-Ag	2	3	2.70	2.77	Ag-Ag	13	16	2.85	2.81
Ag-Ag	1	4	2.70	2.74	Ag-Ag	10	11	2.85	3.00
Ag-Ag	9	10	2.71	2.85	Ag-Ag	7	9	2.87	2.83
Ag-Ag	11	14	2.73	2.78	Ag-Ag	8	9	2.89	2.88
Ag-Ag	1	3	2.73	2.81	Ag-Ag	10	12	2.89	2.98
Ag-Ag	3	8	2.73	2.80	Ag-Ag	6	8	2.92	3.07
Ag-Ag	15	16	2.74	2.82	Ag-Ag	4	8	2.93	3.00
Ag-Ag	4	5	2.74	2.76	Ag-Ag	5	10	2.94	3.07
Ag-Ag	5	8	2.75	2.79	Ag-Ag	3	5	2.96	3.00
Ag-Ag	12	16	2.75	2.86	Ag-Ag	9	13	3.10	3.34
Ag-Ag	1	8	2.75	2.79	Ag-Ag	5	7	3.11	3.34
Ag-Ag	11	13	2.76	2.81	Ag-Ag	4	7	3.12	3.31
Ag-Ag	12	14	2.77	2.83	Ag-Ag	7	12	3.16	3.21
Ag-Ag	5	6	2.78	2.91	Ag-Ag	3	6	3.17	3.34
Ag-Ag	14	15	2.78	2.81	Ag-Ag	11	12	3.21	3.24
Ag-Ag	7	8	2.79	2.80	Ag-Ag	10	14	3.24	3.42
Ag-Ag	6	10	2.79	2.76	Ag-Ag	6	11	3.41	3.38

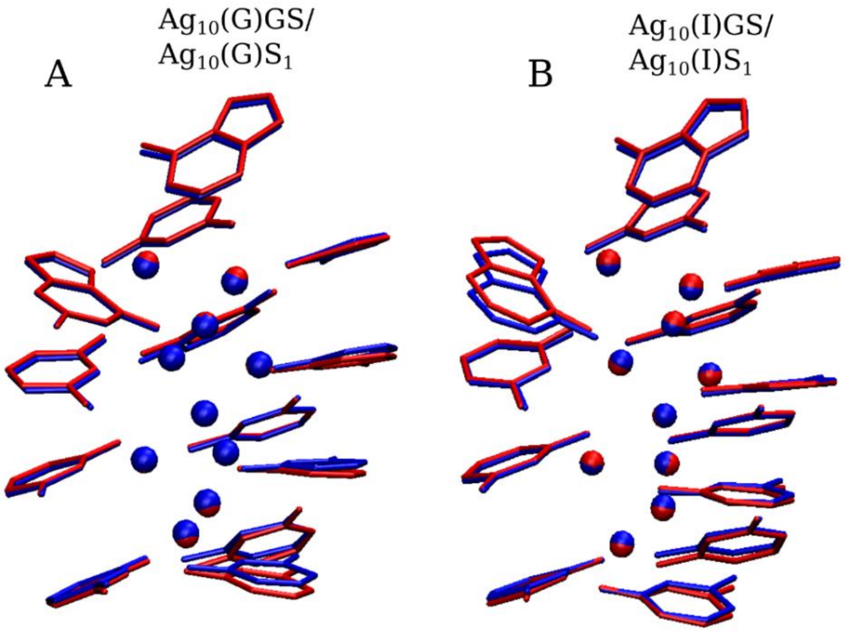

Figure S3. Illustration of A) the molecular structure of the GS (red) and of the S_1 state of Ag_{10}(G) (blue), B) the molecular structure of the GS (red) and of the S_1 state of Ag_{10}(I) (blue).

Table S3. RMSD values of superimposed GS and S_1 geometries of Ag_{16}(G) and Ag_{16}(I).

	Ag_{10} S_0 /Ag_{10}(G) S_1		Ag_{10}(I) S_0 / Ag_{10}(I) S_1	
	Ag_N	Full	Ag_N	Full
RMSD, Å	0.08	4.41	0.06	4.23

Table S4. Coefficients of the most important orbitals excitations of the S_1 and T_1 states calculated using ground state geometries. Only those more than 0.1 are shown.

	**Ag10(G)**		**Ag16(G)**	
T_1			H-1 → L	-0.10
	H → L	0.68	H → L	0.68
T_2			H-3 → L+1	0.18
			H-2 → L+1	0.11
			H-1 → L+1	-0.11
			H → L+1	0.63
S_1	H → L	0.70	H → L	0.70

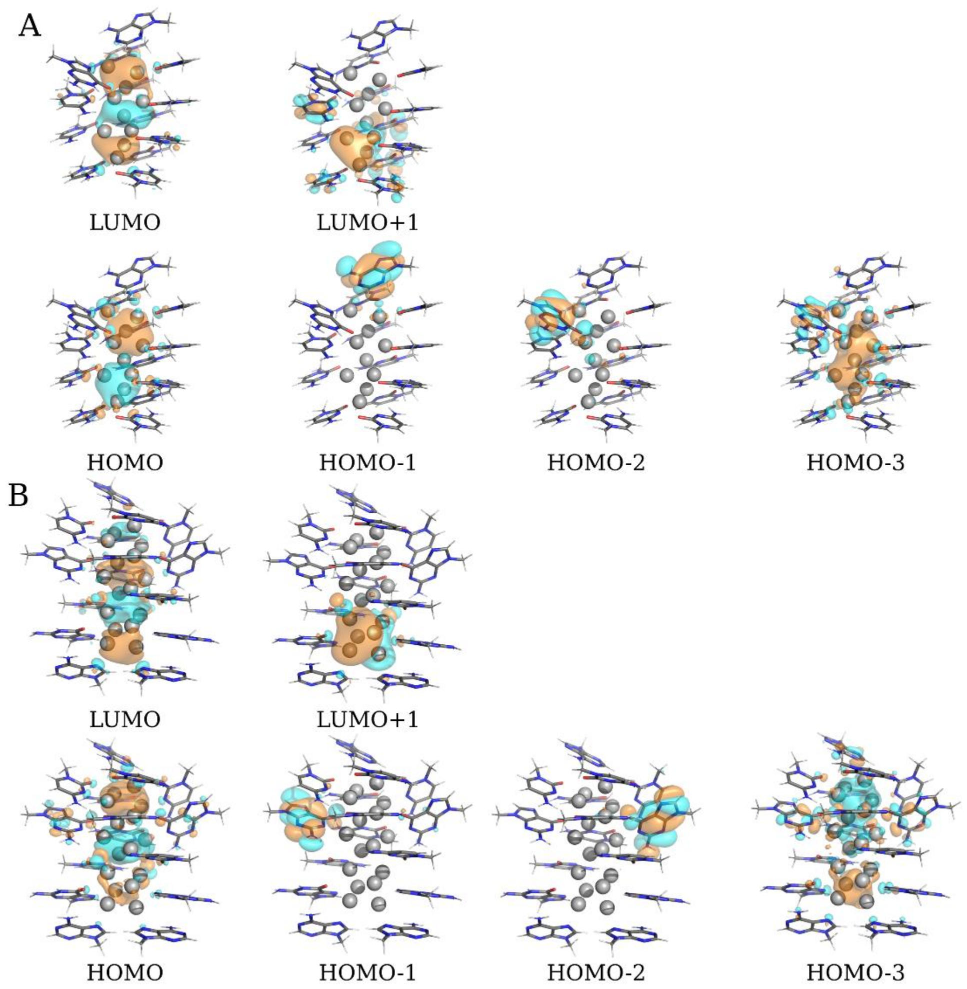

Figure S4. Illustration of the frontier MOs of the A) Ag_{10}(G) and B) Ag_{16}(G) in the ground state.

Table S5. Coefficients of the most important orbitals excitations of the S_1 and T_1 states calculated using S_1 geometries. Only those more than 0.01 are shown.

	**Ag10(G)**		**Ag10(I)**		**Ag16(G)**		**Ag16(I)**	
T_1	H-3 → L	-0.028	H-3 → L	0.030	H-3 → L	-0.073	H-3 → L	-0.062
	H-3 → L+1	0.026	H-3 → L+2	-0.012	H-3 → L+4	-0.011	H-3 → L+4	0.012
	H-3 → L+8	0.011	H-3 → L+15	-0.020	H-3 → L+17	-0.018	H-3 → L+6	-0.011
	H-3 → L+12	0.021	H-3 → L+16	0.019	H → L	0.696	H-3 → L+18	-0.018
	H-3→ L+14	-0.019	H-3 → L+33	0.010			H → L	0.700
	H-3 → L+20	-0.011	H-3 → L+35	0.011				
	H → L	0.700	H → L	0.701				
S_1	H-3 → L	0.012	H-3 → L	0.012	H-3 → L	-0.021	H-3 → L	-0.01
	H-3 → L+6	0.014	H-3 → L+15	-0.014	H-3 → L+17	-0.013	H → L	0.703
	H-3 → L+8	-0.010	H-3 → L+16	0.011	H → L	0.701		
	H → L	0.702	H → L	0.703				

Photophysical calculations

The fluorescence quantum yield from the S_1 state can be obtained as:[17]

$$\varphi_{fl} = \frac{k_r}{k_r + k_{IC} + \sum_i k_{ISC_i}}$$

where k_{ISC_i} is the ISC rate constant between S_1 and energetically lower triplet states T_i, k_r and k_{IC} are the radiative and IC rate constants of the electronic transition from S_1 to S_0, respectively.

- *Radiative rate constants* k_r

The k_r can be estimated using the Strickler–Berg equation:[18]

$$k_r = \frac{1}{1.5} \cdot f \cdot E^2(S_1 \rightarrow S_0),$$

where f is the oscillator strength and E is the energy difference between the two states.

- *Intersystem crossing rate constants* k_{ISC}

The one-electronic spin-orbit coupling operator of the Pauli-Breit Hamiltonian was used for calculating the spin-orbit coupling matrix elements (SOCME) that is needed in the calculation of the intersystem crossing rate constant (k_{ISC}). The excitation energies calculated at the TDDFT level were used as the zeroth-order approximation. The spin-orbit coupling matrix elements $\langle\phi(T_1)|H_{SO}|\phi(S_1)\rangle$ between the singlet S_1 state and the triplet state T_1 state were calculated at the TDDFT/PBE0 level using the MOLSOC program. In the calculation of SOCMEs, the 5 lowest singlet-singlet and singlet-triplet transitions were considered. The intersystem crossing rate constants (k_{ISC}) between S_1 and T_1 states were calculated using the method described in ref.[19] We used the formula:

$$k_{\text{ISC}} = 1.6 \cdot 10^9 \langle\phi(T_1)|H_{\text{SO}}|\phi(S_1)\rangle^2 F_{0n},$$

where $\langle\phi(T_1)|H_{\text{SO}}|\phi(S_1)\rangle$ is the matrix element of the spin-orbit interaction in cm^{-1},

$F_{0n} = y^n \exp(-y)/n!$ is the Franck-Condon factor with an average wavenumber of 1400 cm^{-1}, where the Huang-Rhys factor y is equal to 0.3,

$1.6 \cdot 10^9$ is a constant in cm^2/s, which depends on the vibrational relaxation,

$n \approx E(T_1) - E(S_1)$,

where $E(T_1)$ and $E(S_1)$ are the excitation energies of the T_1 and S_1 states in cm^{-1}.

- *Internal conversion rate constants* k_{IC}

For the calculation of k_{IC} we used the X-H approximation:[20,21]

$$k_{IC}(p \to q) = 1.6 \cdot 10^9 \langle \psi_p | \Lambda | \psi_q \rangle^2,$$

$$\langle \psi_p | \Lambda | \psi_q \rangle^2 = \left(\sum_{\alpha}^{N_{XH}} \left(\sum_{i,j,a,b} A_{ia}^{p} A_{jb}^{q} \langle i \to a | j \to b \rangle \right)^2 \right) \times N_{XH} \cdot 6.25 \cdot 10^4 \cdot \frac{e^{-E_{pq}/2.17}}{E_{pq}^2},$$

E_{pq} is given in 10^3 cm^{-1}, $\langle \psi_p | \Lambda | \psi_q \rangle$ is the non-adiabatic coupling matrix element (NACME in cm^{-1}) between the total wave functions that include electronic and nuclear parts of the p and q states.

$\sum_{\alpha}^{N_{XH}} \left(\sum_{i,j,a,b} A_{ia}^{p} A_{jb}^{q} \langle i \to a | j \to b \rangle \right)^2$ is the dimensionless overlap of the wave functions of the initial and final states. N_{XH} is the number of accepting X–H modes in molecule. $k_{IC}(p \to q)$ has units of s^{-1}.

Table S6. Cartesian coordinates of the optimized S_1 geometry of the truncated Ag_{10}(G).

244
Ag10C67N43H105O19

Ag	-4.790586000	0.530197000	0.717544000
Ag	-0.855240000	-1.355437000	-0.903400000
Ag	-3.682005000	-1.681682000	-0.591525000
Ag	-2.570131000	0.847906000	-1.144465000
Ag	-2.063476000	-0.177199000	1.352360000
Ag	1.965667000	-1.331566000	-1.225043000
Ag	0.715402000	0.991282000	-0.375265000
Ag	3.616047000	1.188860000	-1.628725000
Ag	0.793894000	-1.145209000	1.399621000
Ag	3.406359000	-0.186363000	0.960674000
N	-0.288291000	-5.630734000	-0.287961000
C	-0.446767000	-4.254427000	-0.223298000
O	-0.084251000	-3.666466000	0.808339000
N	-0.973948000	-3.607878000	-1.294292000
C	-1.359050000	-4.278595000	-2.390708000
N	-1.832906000	-3.591561000	-3.429128000
C	-1.238538000	-5.701165000	-2.455383000
C	-0.685056000	-6.326123000	-1.386022000
H	-2.104421000	-4.077782000	-4.268398000
H	-1.866387000	-2.573447000	-3.434947000
H	-1.547636000	-6.257699000	-3.330139000
H	-0.527561000	-7.398545000	-1.356480000
N	-4.056111000	-5.930947000	-0.026453000
C	-4.007953000	-4.544576000	0.107937000
O	-3.488392000	-4.030964000	1.107292000
N	-4.526971000	-3.779769000	-0.889686000
C	-5.138828000	-4.325971000	-1.949443000
N	-5.667073000	-3.512167000	-2.861886000
C	-5.215800000	-5.745255000	-2.090007000
C	-4.654942000	-6.493693000	-1.109161000
H	-6.171797000	-3.893900000	-3.645068000
H	-5.706183000	-2.507750000	-2.690918000
H	-5.693453000	-6.208286000	-2.943333000
H	-4.654318000	-7.577847000	-1.137492000
N	-2.887336000	0.499010000	-5.480417000
C	-2.573092000	0.403202000	-4.129228000
O	-1.984528000	-0.607975000	-3.727872000
N	-2.935707000	1.433562000	-3.309352000
C	-3.586273000	2.503308000	-3.793141000
N	-3.954960000	3.465625000	-2.946506000
C	-3.899610000	2.614204000	-5.177372000
C	-3.526765000	1.585855000	-5.977491000
H	-4.413831000	4.295392000	-3.284659000
H	-3.690701000	3.431053000	-1.974284000
H	-4.417915000	3.475954000	-5.575786000
H	-3.726864000	1.577302000	-7.042713000
N	-6.626741000	0.840208000	-3.316763000
C	-6.119636000	0.399863000	-2.103309000
O	-5.624799000	-0.742874000	-2.051198000
N	-6.164135000	1.232052000	-1.038058000
C	-6.781625000	2.423116000	-1.117468000
N	-6.832909000	3.180317000	-0.025001000
C	-7.364671000	2.865457000	-2.342688000
C	-7.252857000	2.042578000	-3.413176000
H	-7.321270000	4.060737000	-0.050011000
H	-6.518157000	2.819303000	0.877959000
H	-7.865790000	3.821186000	-2.420647000
H	-7.649057000	2.297160000	-4.389297000

N	-2.535251000	0.370648000	5.597727000
C	-2.493188000	0.407139000	4.200294000
O	-2.593014000	1.479574000	3.599683000
N	-2.343003000	-0.773600000	3.535012000
C	-2.260593000	-1.940148000	4.188369000
N	-2.169377000	-3.076261000	3.501096000
C	-2.282464000	-1.983764000	5.616241000
C	-2.426371000	-0.805571000	6.266921000
H	-2.154859000	-3.957393000	3.986897000
H	-2.249545000	-3.106941000	2.493388000
H	-2.196947000	-2.916752000	6.157597000
H	-2.460735000	-0.740475000	7.349009000
N	-6.346735000	1.386271000	4.602516000
C	-6.003531000	1.278808000	3.262124000
O	-5.954180000	2.294413000	2.551523000
N	-5.706509000	0.051017000	2.768152000
C	-5.749065000	-1.041106000	3.534156000
N	-5.468585000	-2.221474000	2.971362000
C	-6.112910000	-0.953685000	4.911525000
C	-6.396277000	0.282718000	5.393093000
H	-5.370932000	-3.043009000	3.546397000
H	-5.037673000	-2.257436000	2.054733000
H	-6.148417000	-1.825972000	5.550538000
H	-6.668595000	0.454285000	6.428836000
N	2.667175000	-5.333996000	-2.860501000
C	2.687442000	-4.135951000	-2.142229000
O	3.238299000	-4.098527000	-1.038047000
N	2.072457000	-3.049810000	-2.692697000
C	1.482916000	-3.112213000	-3.894337000
N	0.906643000	-2.013962000	-4.380892000
C	1.485447000	-4.322352000	-4.646551000
C	2.084965000	-5.399590000	-4.082389000
H	0.336542000	-2.073694000	-5.207713000
H	0.824094000	-1.176921000	-3.814693000
H	1.016539000	-4.385788000	-5.619015000
H	2.120351000	-6.364964000	-4.574366000
N	5.915616000	-2.517888000	-2.363324000
C	5.157221000	-1.497518000	-1.799638000
O	5.159223000	-1.365985000	-0.568814000
N	4.423691000	-0.696634000	-2.624444000
C	4.403955000	-0.912974000	-3.949911000
N	3.613487000	-0.151073000	-4.713495000
C	5.207785000	-1.928213000	-4.540746000
C	5.932360000	-2.711195000	-3.703241000
H	3.497656000	-0.412910000	-5.680170000
H	2.833778000	0.339121000	-4.283591000
H	5.219151000	-2.092487000	-5.610161000
H	6.550164000	-3.527754000	-4.060149000
N	0.428155000	2.745224000	-4.522602000
C	0.599387000	1.971071000	-3.377018000
O	1.207219000	0.889995000	-3.471578000
N	0.107403000	2.421418000	-2.197008000
C	-0.524275000	3.599661000	-2.138764000
N	-1.060981000	3.975849000	-0.965313000
C	-0.671486000	4.430225000	-3.283340000
C	-0.184933000	3.951489000	-4.456581000
H	-1.407936000	4.915192000	-0.849351000
H	-0.833629000	3.458983000	-0.126667000
H	-1.169903000	5.389010000	-3.233036000
H	-0.263727000	4.502162000	-5.386845000
N	1.140685000	-0.441339000	5.665386000
C	0.862926000	-0.409631000	4.296123000
O	0.593477000	0.665320000	3.764047000

N	0.908904000	-1.586854000	3.606792000
C	1.193740000	-2.741807000	4.220152000
N	1.219749000	-3.855764000	3.482758000
C	1.468033000	-2.784410000	5.619477000
C	1.427604000	-1.606788000	6.293620000
H	1.359010000	-4.744946000	3.933909000
H	0.841556000	-3.845710000	2.536976000
H	1.690620000	-3.712847000	6.128530000
H	1.618235000	-1.543049000	7.359421000
N	4.805891000	-1.196421000	4.998888000
C	4.288347000	-0.630705000	3.829099000
O	3.898414000	0.536391000	3.848660000
N	4.237811000	-1.409849000	2.713327000
C	4.589905000	-2.699801000	2.740702000
N	4.500284000	-3.417472000	1.619060000
C	5.060883000	-3.302821000	3.947986000
C	5.156661000	-2.505929000	5.041650000
H	4.727648000	-4.397683000	1.622214000
H	4.231999000	-2.992892000	0.737994000
H	5.344902000	-4.346155000	3.989705000
H	5.518288000	-2.873477000	5.995678000
N	6.079994000	1.789615000	-0.441015000
C	5.921936000	1.714588000	0.892491000
N	6.614533000	1.001439000	1.771081000
C	7.581462000	0.295983000	1.174855000
C	7.911714000	0.310965000	-0.177827000
C	7.090781000	1.101007000	-1.006025000
N	7.266568000	1.157980000	-2.336092000
N	8.951901000	-0.546815000	-0.445001000
C	9.227500000	-1.070250000	0.727072000
N	8.433710000	-0.609769000	1.743199000
H	5.114855000	2.323502000	1.296951000
H	8.055421000	0.700638000	-2.761397000
H	6.695469000	1.771941000	-2.892343000
H	9.997627000	-1.807494000	0.915108000
C	0.965942000	2.243547000	-5.780953000
H	2.057647000	2.233392000	-5.749046000
H	0.603527000	1.231065000	-5.960273000
H	0.639513000	2.900442000	-6.585918000
C	-2.507660000	-0.608297000	-6.340135000
H	-1.421971000	-0.655446000	-6.445799000
H	-2.869464000	-1.546481000	-5.917994000
H	-2.953237000	-0.457367000	-7.322554000
C	-6.515729000	-0.056527000	-4.454191000
H	-5.501517000	-0.449184000	-4.518649000
H	-7.210964000	-0.893470000	-4.351793000
H	-6.750684000	0.497111000	-5.362804000
C	-6.626553000	2.703385000	5.155359000
H	-7.646284000	2.736457000	5.544884000
H	-5.922333000	2.932084000	5.957970000
H	-6.516548000	3.430884000	4.356033000
C	-2.723812000	1.605842000	6.341681000
H	-3.683615000	1.591437000	6.864020000
H	-1.924851000	1.731795000	7.075483000
H	-2.704628000	2.430556000	5.633250000
C	1.149801000	0.835311000	6.359390000
H	0.200742000	1.349674000	6.212165000
H	1.314305000	0.659929000	7.422517000
H	1.946509000	1.466016000	5.958257000
C	4.888634000	-0.336213000	6.166781000
H	3.902870000	0.051700000	6.425381000
H	5.285996000	-0.912876000	7.001514000
H	5.547772000	0.509379000	5.960964000

C	3.310545000	-6.497692000	-2.266422000
H	2.991286000	-6.603580000	-1.230072000
H	3.026396000	-7.385095000	-2.831394000
H	4.397250000	-6.385708000	-2.287434000
C	0.342087000	-6.268732000	0.855188000
H	-0.238035000	-6.078756000	1.760092000
H	0.391133000	-7.342522000	0.676786000
H	1.354080000	-5.880087000	0.988290000
C	-3.451104000	-6.778848000	0.997635000
H	-4.220135000	-7.375642000	1.492812000
H	-2.715895000	-7.444730000	0.543546000
H	-2.970074000	-6.132071000	1.727380000
C	6.566907000	-3.455376000	-1.458104000
H	6.952806000	-2.904892000	-0.603406000
H	5.846897000	-4.204255000	-1.118257000
H	7.389009000	-3.939085000	-1.986004000
C	8.464789000	-0.995688000	3.137116000
H	7.522479000	-0.695530000	3.594613000
H	8.577983000	-2.077494000	3.220162000
H	9.287541000	-0.502786000	3.661235000
N	1.478938000	5.270473000	0.779994000
C	1.618594000	6.509146000	0.204495000
N	1.147234000	7.570947000	0.857443000
N	2.195567000	6.673803000	-0.981003000
C	2.638017000	5.531772000	-1.522967000
C	2.554516000	4.239646000	-1.006920000
C	1.870756000	4.049801000	0.223044000
O	1.574278000	2.998666000	0.811409000
N	3.125145000	3.351686000	-1.892503000
C	3.535344000	4.083431000	-2.903289000
N	3.263368000	5.410375000	-2.731332000
H	1.274389000	8.480460000	0.406382000
H	0.716615000	7.498957000	1.763113000
H	4.036200000	3.708579000	-3.784688000
H	0.996472000	5.188625000	1.667170000
C	3.510810000	6.483278000	-3.674525000
H	3.760934000	7.389233000	-3.121795000
H	4.348658000	6.209699000	-4.315065000
H	2.622985000	6.663058000	-4.285404000
O	1.873995000	9.500069000	-1.068758000
H	2.721772000	9.875898000	-0.806854000
H	2.103170000	8.614670000	-1.407590000
O	-0.252737000	7.735921000	3.271833000
H	-1.000910000	8.059419000	2.725629000
H	-0.380341000	6.777635000	3.383118000
O	-0.751726000	10.256106000	-0.434667000
H	-1.259744000	10.079167000	-1.232679000
H	0.171459000	10.074296000	-0.688357000
O	-0.001735000	4.993363000	3.157569000
H	-0.625422000	4.354077000	2.754551000
H	0.649173000	4.413708000	3.606843000
O	1.872914000	3.082420000	3.571420000
H	1.556006000	2.205684000	3.862175000
H	1.887222000	2.990512000	2.599403000
O	-1.129847000	2.890901000	1.853441000
H	-0.279167000	2.438763000	1.926595000
H	-1.728530000	2.365970000	2.428560000
O	-2.070831000	8.803976000	1.504414000
H	-1.532651000	9.284449000	0.837582000
H	-2.568725000	9.497209000	1.948673000

Table S7. Cartesian coordinates of the optimized S_1 geometry of the truncated Ag_{16}(G).

292
Ag16C82N59H115O18Cl2

Ag	-2.678923000	1.181201000	0.962002000
Ag	0.406399000	0.930770000	1.054409000
Ag	3.876099000	1.033182000	1.358495000
Ag	2.408825000	1.171246000	-0.986411000
Ag	-5.570845000	-1.096738000	-0.748063000
Ag	-2.611508000	-0.917664000	-0.911921000
Ag	0.413755000	-0.868890000	-1.147352000
Ag	3.753241000	-1.265999000	-1.353684000
Ag	-4.189057000	-1.044605000	1.673498000
Ag	-1.026147000	-1.511023000	1.384374000
Ag	2.307302000	-1.251585000	0.965781000
Ag	5.453767000	-1.320818000	0.926525000
Ag	-5.738032000	1.243664000	0.848678000
Ag	-4.265130000	1.260382000	-1.628376000
Ag	-1.039670000	1.606888000	-1.387028000
Ag	5.636619000	0.812711000	-0.851230000
Cl	1.082343000	-1.241096000	3.241554000
Cl	1.091708000	1.243134000	-3.248907000
N	-6.802783000	-4.691915000	1.895012000
C	-6.116075000	-3.542462000	1.533555000
O	-6.368948000	-3.035221000	0.420099000
N	-5.210853000	-3.014533000	2.384889000
C	-4.936292000	-3.625205000	3.551265000
N	-4.037348000	-3.082918000	4.365271000
C	-5.594882000	-4.836172000	3.921304000
C	-6.518408000	-5.325129000	3.062379000
H	-3.745751000	-3.590889000	5.184005000
H	-3.486901000	-2.272167000	4.085983000
H	-5.373286000	-5.340486000	4.852534000
H	-7.076396000	-6.233284000	3.259816000
N	-7.942412000	0.311033000	0.337434000
C	-8.464021000	0.561929000	-0.883375000
N	-9.629429000	0.173836000	-1.375276000
C	-10.321193000	-0.564537000	-0.491804000
C	-9.911592000	-0.911321000	0.793645000
C	-8.656169000	-0.427514000	1.219510000
N	-8.183770000	-0.664678000	2.444072000
N	-10.857953000	-1.674746000	1.433160000
C	-11.815048000	-1.781988000	0.542659000
N	-11.554871000	-1.140214000	-0.640308000
H	-7.827585000	1.154738000	-1.535722000
H	-7.261397000	-0.348176000	2.751638000
H	-8.728986000	-1.245297000	3.060792000
H	-12.740718000	-2.325448000	0.683701000
N	-6.334437000	-1.176070000	-5.115393000
C	-6.085994000	-0.857914000	-3.780173000
O	-5.787133000	0.310341000	-3.494557000
N	-6.188595000	-1.841901000	-2.849200000
C	-6.509240000	-3.096190000	-3.204067000
N	-6.650235000	-4.013879000	-2.250864000
C	-6.705535000	-3.447468000	-4.572569000
C	-6.613361000	-2.450685000	-5.484539000
H	-6.858485000	-4.966678000	-2.498680000
H	-6.539119000	-3.767108000	-1.267354000
H	-6.941803000	-4.460533000	-4.869793000
H	-6.765991000	-2.618508000	-6.544664000
N	-2.118630000	-0.206563000	-5.381103000
C	-2.393723000	-0.150433000	-4.021794000

O	-2.472431000	0.977377000	-3.480903000
N	-2.552592000	-1.294980000	-3.325849000
C	-2.563471000	-2.481737000	-3.960784000
N	-2.718086000	-3.589740000	-3.244706000
C	-2.415418000	-2.553698000	-5.380923000
C	-2.171859000	-1.396394000	-6.038093000
H	-2.696357000	-4.482478000	-3.710037000
H	-2.775770000	-3.559575000	-2.214712000
H	-2.456572000	-3.497683000	-5.908134000
H	-2.004470000	-1.356624000	-7.108413000
N	8.644525000	-5.334831000	2.563505000
C	9.285476000	-5.888373000	1.528610000
N	9.409758000	-5.431843000	0.284964000
C	8.766462000	-4.272086000	0.130102000
C	8.039460000	-3.580549000	1.099155000
C	8.012085000	-4.162746000	2.384712000
N	7.374338000	-3.615956000	3.433074000
N	7.508726000	-2.427508000	0.557161000
C	7.913993000	-2.435277000	-0.697932000
N	8.671716000	-3.516926000	-1.012907000
H	9.774435000	-6.837994000	1.739182000
H	7.480280000	-4.048098000	4.336730000
H	7.002902000	-2.682643000	3.384951000
H	7.669350000	-1.673391000	-1.426159000
N	1.180003000	-5.081004000	-1.808739000
C	1.151927000	-3.770388000	-1.335474000
O	1.230720000	-3.563746000	-0.118984000
N	1.027989000	-2.765655000	-2.244726000
C	1.068257000	-3.004277000	-3.561448000
N	1.031626000	-1.960660000	-4.391828000
C	1.140635000	-4.337886000	-4.059108000
C	1.175288000	-5.336985000	-3.140720000
H	1.050701000	-2.099230000	-5.388551000
H	1.020480000	-1.009564000	-4.032934000
H	1.149479000	-4.551456000	-5.119578000
H	1.207076000	-6.382637000	-3.425302000
N	-6.461166000	5.152999000	-1.795238000
C	-5.910361000	3.916617000	-1.480779000
O	-6.259677000	3.370520000	-0.414046000
N	-5.030406000	3.348807000	-2.333024000
C	-4.668952000	3.977398000	-3.463418000
N	-3.800291000	3.382990000	-4.277351000
C	-5.215347000	5.251296000	-3.802647000
C	-6.100766000	5.796036000	-2.935287000
H	-3.424137000	3.901880000	-5.053974000
H	-3.320275000	2.525095000	-4.002829000
H	-4.935549000	5.763645000	-4.713360000
H	-6.565172000	6.760587000	-3.104984000
N	-5.580914000	1.857705000	5.269677000
C	-5.706727000	1.402049000	3.960051000
O	-5.796306000	0.178430000	3.748241000
N	-5.723662000	2.312007000	2.954609000
C	-5.563276000	3.623005000	3.199780000
N	-5.579406000	4.468603000	2.173044000
C	-5.393521000	4.102881000	4.533738000
C	-5.422664000	3.183938000	5.526260000
H	-5.200277000	5.399047000	2.266841000
H	-5.677398000	4.107332000	1.224281000
H	-5.268406000	5.156368000	4.745449000
H	-5.324182000	3.453508000	6.572004000
N	-1.898975000	0.374811000	5.388542000
C	-2.289035000	0.356574000	4.056514000
O	-2.555236000	-0.750648000	3.533856000

N	-2.343693000	1.513719000	3.363612000
C	-2.117453000	2.689981000	3.978017000
N	-2.169257000	3.805539000	3.260641000
C	-1.824861000	2.741003000	5.376945000
C	-1.707011000	1.560465000	6.027654000
H	-2.016903000	4.691084000	3.715345000
H	-2.371901000	3.787545000	2.247484000
H	-1.671443000	3.680875000	5.890414000
H	-1.454241000	1.495648000	7.079931000
N	9.180086000	4.704383000	-1.910025000
C	9.830690000	5.063775000	-0.798057000
N	9.883331000	4.438200000	0.375558000
C	9.148691000	3.323602000	0.360072000
C	8.399697000	2.826373000	-0.707302000
C	8.455392000	3.573294000	-1.903219000
N	7.800940000	3.227817000	-3.027186000
N	7.773950000	1.652380000	-0.338451000
C	8.144730000	1.460381000	0.912528000
N	8.967844000	2.429129000	1.386530000
H	10.396917000	5.990696000	-0.870553000
H	8.002480000	3.747179000	-3.866963000
H	7.396457000	2.311394000	-3.123286000
H	7.828733000	0.627217000	1.526304000
N	1.629572000	5.047490000	1.607405000
C	1.508339000	3.731359000	1.169202000
O	1.567577000	3.490623000	-0.044067000
N	1.318546000	2.757848000	2.099932000
C	1.412197000	3.020879000	3.410616000
N	1.313567000	2.003794000	4.267514000
C	1.604193000	4.355149000	3.874056000
C	1.683498000	5.330404000	2.933115000
H	1.348389000	2.166962000	5.260250000
H	1.205169000	1.048834000	3.935673000
H	1.664113000	4.589394000	4.928457000
H	1.800902000	6.376383000	3.192930000
C	-7.784318000	-5.251053000	0.973020000
H	-7.295894000	-5.906621000	0.246938000
H	-8.515476000	-5.823770000	1.543644000
H	-8.286949000	-4.438249000	0.452911000
C	-5.614377000	0.911031000	6.379698000
H	-5.709389000	-0.090007000	5.966285000
H	-4.692751000	0.986397000	6.960837000
H	-6.469073000	1.126138000	7.024779000
C	-1.821150000	1.025323000	-6.104756000
H	-1.247602000	1.686722000	-5.456900000
H	-1.230684000	0.777035000	-6.986841000
H	-2.741947000	1.526493000	-6.416151000
C	-1.747005000	-0.879008000	6.119964000
H	-1.432645000	-1.657372000	5.426166000
H	-0.981710000	-0.749416000	6.886122000
H	-2.689091000	-1.169093000	6.593988000
C	1.717853000	6.090907000	0.596301000
H	1.696544000	7.061442000	1.090455000
H	2.643141000	5.990444000	0.025385000
H	0.873171000	6.009504000	-0.089283000
C	9.552805000	2.508012000	2.710151000
H	9.074329000	1.768356000	3.351410000
H	10.625949000	2.310757000	2.664076000
H	9.387330000	3.505319000	3.120563000
C	9.266680000	-3.830560000	-2.298034000
H	10.065211000	-3.124108000	-2.533082000
H	9.681613000	-4.836145000	-2.233068000
H	8.505795000	-3.801437000	-3.079979000

C	-12.440424000	-1.047899000	-1.785769000
H	-13.025587000	-1.964499000	-1.861878000
H	-13.112302000	-0.191749000	-1.687368000
H	-11.837607000	-0.928935000	-2.686596000
C	-7.381976000	5.770877000	-0.849057000
H	-8.085776000	5.023104000	-0.485951000
H	-6.832628000	6.189244000	-0.001925000
H	-7.923457000	6.568222000	-1.357235000
C	-6.243888000	-0.109590000	-6.100587000
H	-6.825209000	0.748609000	-5.763550000
H	-5.205710000	0.201356000	-6.236565000
H	-6.641345000	-0.471815000	-7.048157000
C	1.212484000	-6.149083000	-0.822204000
H	1.191626000	-7.106982000	-1.340575000
H	2.118723000	-6.078141000	-0.218541000
H	0.346452000	-6.069533000	-0.162542000
N	5.143436000	-5.415135000	1.064859000
C	5.564075000	-6.595030000	0.386326000
N	5.876302000	-7.570447000	1.328004000
N	5.597373000	-6.649453000	-0.996452000
C	5.197995000	-5.466663000	-1.501250000
C	4.775789000	-4.282845000	-0.912391000
C	4.758249000	-4.205779000	0.505225000
O	4.469909000	-3.262433000	1.268975000
N	4.466898000	-3.330420000	-1.858253000
C	4.697344000	-3.892715000	-3.017302000
N	5.132425000	-5.175426000	-2.881302000
H	5.111865000	-5.314200000	2.132183000
H	6.366397000	-8.531669000	1.249733000
H	5.983526000	-7.330639000	2.366570000
H	4.555485000	-3.427827000	-3.982957000
N	-1.825782000	-3.621547000	1.543807000
C	-1.644265000	-4.467367000	2.600916000
N	-0.916968000	-3.967764000	3.652027000
N	-2.137851000	-5.708122000	2.717434000
C	-2.861325000	-6.066246000	1.638853000
C	-3.098758000	-5.304697000	0.487671000
C	-2.533801000	-4.001424000	0.445625000
O	-2.670172000	-3.228650000	-0.546813000
N	-3.878086000	-5.986329000	-0.422208000
C	-4.112789000	-7.131583000	0.158148000
N	-3.532202000	-7.259696000	1.401197000
H	-0.518385000	-4.588539000	4.444323000
H	-0.327788000	-3.184773000	3.404185000
H	-4.697899000	-7.941416000	-0.260116000
N	5.623146000	5.095913000	-1.253125000
C	6.131453000	6.255484000	-0.574958000
N	6.548498000	7.269045000	-1.416693000
N	6.156332000	6.310510000	0.779917000
C	5.659294000	5.167307000	1.301919000
C	5.139767000	4.002726000	0.723587000
C	5.136566000	3.922078000	-0.689656000
O	4.810214000	3.066499000	-1.515499000
N	4.764249000	3.083143000	1.684158000
C	5.049679000	3.657363000	2.826077000
N	5.575766000	4.899711000	2.678638000
H	5.599105000	4.931992000	-2.273305000
H	7.073701000	8.165740000	-1.123586000
H	6.706887000	7.129814000	-2.466642000
H	4.885675000	3.218701000	3.800493000
N	-1.653776000	3.804363000	-1.487051000
C	-1.375701000	4.642116000	-2.522833000
N	-0.640763000	4.117100000	-3.546174000

N	-1.796410000	5.914898000	-2.631544000
C	-2.559456000	6.318310000	-1.595305000
C	-2.890513000	5.557397000	-0.472276000
C	-2.373892000	4.229567000	-0.408470000
O	-2.563803000	3.477967000	0.587847000
N	-3.692425000	6.270385000	0.394442000
C	-3.841188000	7.430087000	-0.187010000
N	-3.182053000	7.538823000	-1.390088000
H	-0.179599000	4.725585000	-4.309552000
H	-0.116822000	3.285342000	-3.302681000
H	-4.416342000	8.260310000	0.202616000
C	-3.203478000	8.696309000	-2.255785000
H	-3.582460000	9.551114000	-1.696232000
H	-2.193840000	8.917662000	-2.598867000
H	-3.843333000	8.518791000	-3.124193000
C	5.475667000	-6.035272000	-3.989263000
H	5.066318000	-5.614442000	-4.908507000
H	6.563238000	-6.122738000	-4.078784000
H	5.053757000	-7.023552000	-3.817840000
C	-3.674102000	-8.403492000	2.272732000
H	-3.874707000	-9.291379000	1.672290000
H	-2.750500000	-8.547894000	2.826509000
H	-4.490583000	-8.254296000	2.985031000
C	5.982930000	5.733529000	3.787108000
H	5.661984000	5.259582000	4.715813000
H	7.069169000	5.845795000	3.794223000
H	5.523707000	6.715862000	3.695109000
H	-1.301125000	-6.651577000	4.265116000
H	-1.449046000	-6.448653000	5.772163000
O	-0.782183000	-6.517659000	5.079993000
H	1.007544000	-6.108962000	5.133982000
O	1.931979000	-5.809882000	5.058785000
H	2.358421000	-6.480726000	4.516263000
H	2.300952000	-4.130012000	4.434427000
O	2.748010000	-3.531924000	3.796002000
H	2.097131000	-2.845043000	3.621684000
H	1.936734000	3.224350000	-3.727660000
O	2.669045000	3.840845000	-3.825088000
H	2.323847000	4.529421000	-4.434890000
O	2.178435000	6.279727000	-4.947929000
H	2.610622000	6.938373000	-4.395107000
H	1.271987000	6.617584000	-5.065780000
O	-0.540202000	6.897857000	-4.963356000
H	-1.218798000	6.780940000	-5.637452000
H	-1.045302000	6.995195000	-4.134707000

References

(1) Cerretani, C.; Kanazawa, H.; Vosch, T.; Kondo, J. Crystal Structure of a NIR-Emitting DNA-Stabilized Ag16 Nanocluster. *Angew. Chem., Int. Ed.* **2019**, *58* (48), 17153–17157.

(2) Abraham, M. J.; Murtola, T.; Schulz, R.; Páll, S.; Smith, J. C.; Hess, B.; Lindah, E. GROMACS: High Performance Molecular Simulations through Multi-Level Parallelism from Laptops to Supercomputers. *SoftwareX* **2015**, *1–2*, 19–25.

(3) Hutter, J.; Iannuzzi, M.; Schiffmann, F.; Vandevondele, J. Cp2k: Atomistic Simulations of Condensed Matter Systems. *WIREs Comput. Mol. Sci.* **2014**, *4* (1), 15–25.

(4) Laino, T.; Mohamed, F.; Laio, A.; Parrinello, M. An Efficient Real Space Multigrid QM/MM Electrostatic Coupling. *J. Chem. Theory. Comput.* **2005**, *1* (6), 1176–1184.

(5) Lippert, G.; Hutter, J.; Parrinello, M. A Hybrid Gaussian and Plane Wave Density Functional Scheme. *Mol. Phys.* **1997**, *92* (3), 477–488.

(6) VandeVondele, J.; Hutter, J. Gaussian Basis Sets for Accurate Calculations on Molecular Systems in Gas and Condensed Phases. *J. Chem. Phys.* **2007**, *127* (11), 114105.

(7) VandeVondele, J.; Krack, M.; Mohamed, F.; Parrinello, M.; Chassaing, T.; Hutter, J. Quickstep: Fast and Accurate Density Functional Calculations Using a Mixed Gaussian and Plane Waves Approach. *Comput. Phys. Commun.* **2005**, *167* (2), 103–128.

(8) Goedecker, S.; Teter, M. Separable Dual-Space Gaussian Pseudopotentials. *Phys. Rev. B* **1996**, *54* (3), 1703.

(9) Hartwigsen, C.; Goedecker, S.; Hutter, J. Relativistic Separable Dual-Space Gaussian Pseudopotentials from H to Rn. *Phys. Rev. B* **1998**, *58* (7), 3641.

(10) Adamo, C.; Barone, V. Toward Reliable Density Functional Methods without Adjustable Parameters: The PBE0 Model. *J. Chem. Phys.* **1999**, *110* (13), 6158–6170.

(11) Grimme, S. Supramolecular Binding Thermodynamics by Dispersion-Corrected Density Functional Theory. *Chem. Eur. J.* **2012**, *18* (32), 9955–9964.

(12) Guidon, M.; Hutter, J.; Vandevondele, J. Auxiliary Density Matrix Methods for Hartree−Fock Exchange Calculations. *J. Chem. Theory Comput.* **2010**, *6* (8), 2348–2364.

(13) Frisch, M. J.; Trucks, G. W.; Schlegel, H. B.; Scuseria, G. E.; Robb, M. A.; Cheeseman, J. R.; Scalmani, G.; Barone, V.; Petersson, G. A.; Nakatsuji, H.; Li, X.; Caricato, M.; Marenich, A. V; Bloino, J.; Janesko, B. G.; Gomperts, R.; Mennucci, B.; Hratchian, H. P.; Ortiz, J. V; Izmaylov, A. F.; Sonnenberg, J. L.; Williams-Young, D.; Ding, F.; Lipparini, F.; Egidi, F.; Goings, J.; Peng, B.; Petrone, A.;

Henderson, T.; Ranasinghe, D.; Zakrzewski, V. G.; Gao, J.; Rega, N.; Zheng, G.; Liang, W.; Hada, M.; Ehara, M.; Toyota, K.; Fukuda, R.; Hasegawa, J.; Ishida, M.; Nakajima, T.; Honda, Y.; Kitao, O.; Nakai, H.; Vreven, T.; Throssell, K.; Montgomery Jr., J. A.; Peralta, J. E.; Ogliaro, F.; Bearpark, M. J.; Heyd, J. J.; Brothers, E. N.; Kudin, K. N.; Staroverov, V. N.; Keith, T. A.; Kobayashi, R.; Normand, J.; Raghavachari, K.; Rendell, A. P.; Burant, J. C.; Iyengar, S. S.; Tomasi, J.; Cossi, M.; Millam, J. M.; Klene, M.; Adamo, C.; Cammi, R.; Ochterski, J. W.; Martin, R. L.; Morokuma, K.; Farkas, O.; Foresman, J. B.; Fox, D. J. Gaussian09 Revision A.01. *Gaussian, Inc., Wallingford CT, 2009* **2016**.

(14) Tomasi, J.; Mennucci, B.; Cammi, R. Quantum Mechanical Continuum Solvation Models. *Chem. Rev.* **2005**, *105* (8), 2999–3093.

(15) Petersson, G. A.; Bennett, A.; Tensfeldt, T. G.; Al-Laham, M. A.; Shirley, W. A.; Mantzaris, J. A Complete Basis Set Model Chemistry. I. The Total Energies of Closed-Shell Atoms and Hydrides of the First-Row Elements. *J. Chem. Phys.* **1988**, *89* (4), 2193–2218.

(16) Schäfer, A.; Huber, C.; Ahlrichs, R. Fully Optimized Contracted Gaussian Basis Sets of Triple Zeta Valence Quality for Atoms Li to Kr. *J. Chem. Phys.* **1994**, *100* (8), 5829–5835.

(17) P.S. Molecular Spectroscopy of the Triplet State: S.P. McGlynn, T. Azumi and M. Kkinoshita, *J. Mol. Struct.* **1972**, *12* (2), 310.

(18) Strickler, S. J.; Berg, R. A.; Strickler, S. J.; Berg, R. A. Relationship between Absorption Intensity and Fluorescence Lifetime of Molecules. *J. Chem. Phys.* **1962**, *37* (4), 814–822.

(19) Valiev, R. R.; Cherepanov, V. N.; Baryshnikov, G. V.; Sundholm, D. First-Principles Method for Calculating the Rate Constants of Internal-Conversion and Intersystem-Crossing Transitions. *Phys. Chem. Chem. Phys.* **2018**, *20* (9), 6121–6133.

(20) Valiev, R. R.; Nasibullin, R. T.; Cherepanov, V. N.; Kurtsevich, A.; Sundholm, D.; Kurtén, T. Fast Estimation of the Internal Conversion Rate Constant in Photophysical Applications. *Phys. Chem. Chem. Phys.* **2021**, *23* (11), 6344–6348.

(21) Valiev, R. R.; Nasibullin, R. T.; Merzlikin, B. S.; Khoroshkin, K.; Cherepanov, V. N.; Sundholm, D. Internal Conversion Induced by External Electric and Magnetic Fields. *Phys. Chem. Chem. Phys.* **2024**, *26* (4), 2945–2950.
